# A subtype of laminopathies: Generalized lipodystrophy‐associated progeroid syndrome caused by LMNA gene c.29C>T mutation

**DOI:** 10.1111/jdi.14055

**Published:** 2023-07-13

**Authors:** Shipeng Huang, Yan Zhang, Zuan Zhan, Shuhao Gong

**Affiliations:** ^1^ Department of Emergency First Affiliated Hospital of Nanchang University Nanchang China; ^2^ Department of Endocrinology and Metabolism First Affiliated Hospital of Nanchang University Nanchang China; ^3^ Jiangxi Clinical Research Center for Endocrine and Metabolic Disease Nanchang China; ^4^ Jiangxi Branch of National Clinical Research Center for Metabolic Disease Nanchang China

**Keywords:** Congenital Generalized, Diabetes Mellitus, Laminopathies, Lipoatrophic, Lipodystrophy, Progeria Syndrome

## Abstract

The term laminopathies refers to a group of congenital diseases characterized by accelerated degeneration of human tissues. Mutations in LMNA, LMNB, ZMPSTE24, and other genes lead to structural and functional abnormalities associated with lamins. One subtype of laminopathy is the generalized lipodystrophy‐associated progeroid syndrome (GLPS), which occurs in patients with heterozygous mutations of the LMNA gene c.29C>T(p.T10I). This paper reports the first case of GLPS in China and compares the clinical features of other GLPS patients with literature reports. A 16‐year‐old male patient was treated for diabetic ketoacidosis, presenting with premature aging appearance, systemic lipodystrophy, severe fatty liver, and decreased bone density. After peripheral blood DNA extraction and second‐generation sequencing, a heterozygous mutation of exon 1 of the LMNA gene c.29C>T(p.T10I) was detected. This case of GLPS may provide a diagnostic and therapeutic basis for potential patients.

## Graphical abstract

This paper reports the first case of generalized lipodystrophy‐associated progeroid syndrome (GLPS) with LMNA c.29C>T mutation in China.

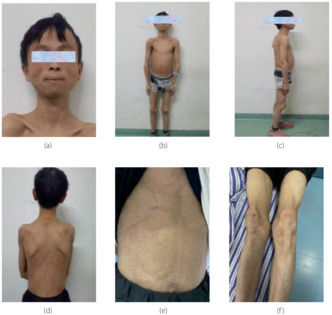

Laminopathy is a rare disease affecting multiple tissues and organs, such as the skin, fat, bone, muscle, and cardiovascular system. LMNA is the most common pathogenic gene causing laminopathies, and the clinical phenotypes caused by different mutation sites vary. Hussain *et al*.^1^ summarized the clinical features of 10 patients with LMNA c.29C>T (p.T10I) gene mutation and categorized it as a subtype of laminopathy characterized by generalized lipodystrophy‐associated progeroid syndrome (GLPS). Currently, there are no research data on Chinese patients with GLPS, and this report is the first case of a Chinese patient with GLPS.

## ONSET OF DISEASE

The patient is a 16‐year‐old Han male who was admitted to the hospital in 2022, due to abdominal pain, nausea, and vomiting for 3 days. Diabetic ketoacidosis was diagnosed in the emergency department.

## MEDICAL HISTORY

He was born at term in a non‐consanguineous family without a history of dystocia. His birth weight was 3.5 kg, but his height and weight development had been lagging behind his peers. The patient's parents and two sisters were in good health, and there were no similar patients in the family.

## PHYSICAL EXAMINATION

**Figure 1:**
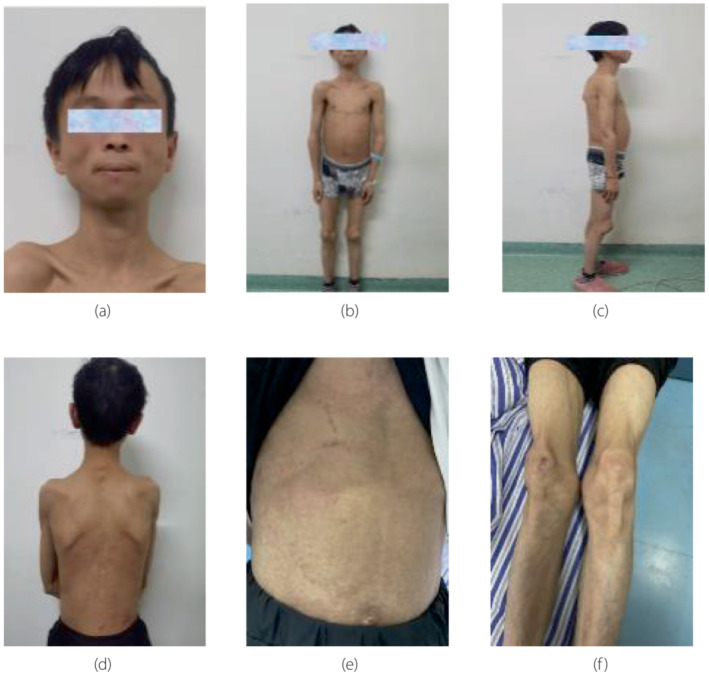
Progeria face (a), subcutaneous fat in limbs and trunk is thin and muscle is atrophic (b–d), abdominal distention with papules (e), contracture of both knees (f).

The patient's height was 144 cm and weight was 25 kg, BMI of 12.06 kg/m^2^. He presented with a progeria face (Figure 1a), thinning and soft hair, sunken cheeks, and thin subcutaneous fat on the limbs, trunk, and buttocks (Figure 1b–d). There were black and white papules on the abdomen (Figure 1e), but no signs of acanthosis nigricans. Abdominal distention was present, and a palpable mass was found 10 cm below the liver and ribs. The patient showed atrophy of the limbs, normal muscle strength, and tension of limbs, knee contracture (Figure 1f), and limited squat to knee curvature of 90°. The external genitalia showed normal development, and no other abnormalities were observed during the physical examination.

## LABORATORY TESTS AND IMAGING EXAMINATIONS

**Figure 2:**
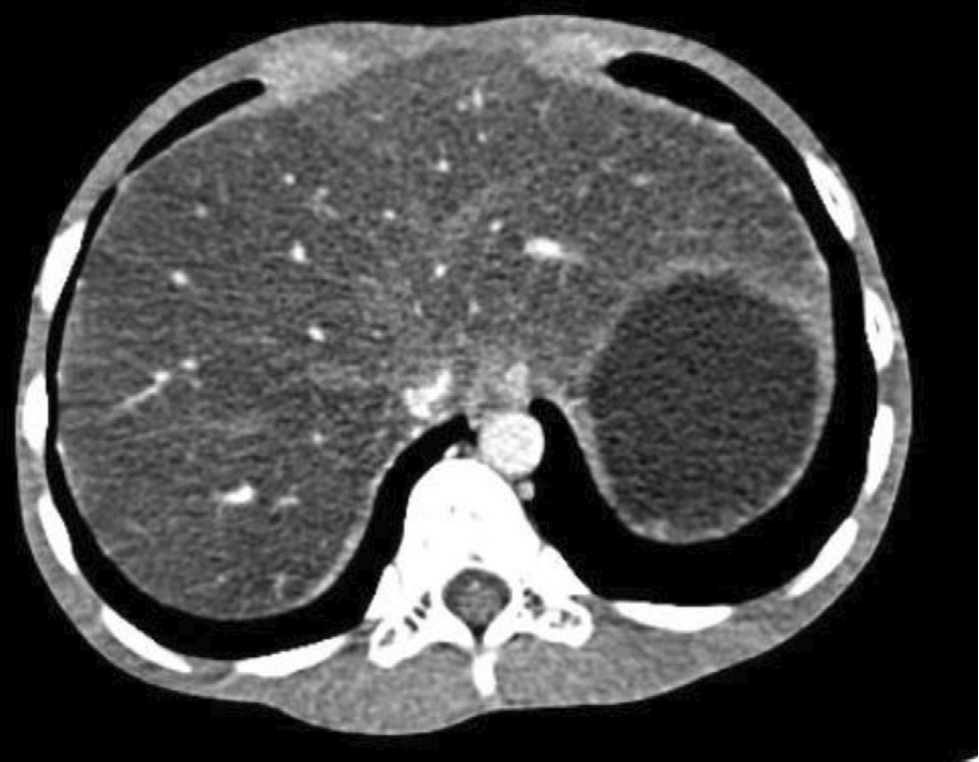
Abdominal CT: increased liver volume and decreased liver parenchymal density and very little subcutaneous fat layer.

The patient's blood lipid levels were high, with triglyceride levels of 49.28 mmol/L (0–1.7), total cholesterol levels of 25.63 mmol/L (0–5.7), low density lipoprotein cholesterol levels of 16.74 mmol/L (0–3.62), and high density lipoprotein cholesterol levels of 0.34 mmol/L (1.16–1.42). The HbA1c was 15.9%. The level of 25‐hydroxy vitamin D3 was 16.9 ng/mL (>20 ng/mL). The abdominal CT (Figure 2) showed (1) severe fatty liver with minimal subcutaneous fat layer; (2) T6 vertebral body compressibility changes, indicative of osteoporosis.

## GENE SEQUENCING AND PEDIGREE VALIDATION

**Figure 3:**
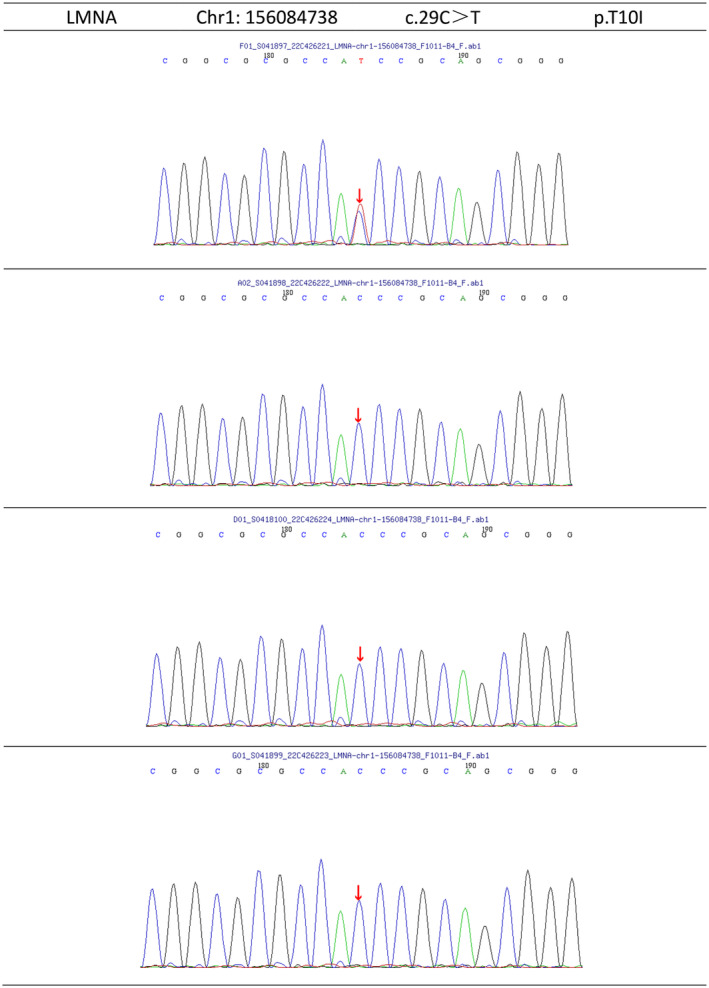
Gene sequencing results: from top to bottom, the sequencing results of the proband, father, mother, and sister, respectively, the arrow indicates the mutation base.

Whole‐exon gene second‐generation sequencing and the gene sequences of the family members were verified by Sanger sequencing (Beijing MyGenostics Co., Ltd, Beijing, China). The sequencing results (Figure 3) showed a heterozygous mutation of c.29C>T in exon 1 of the LMNA gene, resulting in a change of threonine 10 to isoleucine (p.T10I). The family verification analysis showed it was a spontaneous mutation.

## CLINICAL DIAGNOSIS AND TREATMENT FOLLOW‐UP

Laminopathies include clinical phenotypes such as atypical progeria, insulin‐resistant diabetes mellitus, generalized lipodystrophy, severe fatty liver, and diffuse black and white dermatoid papules.

The treatment process is shown in Table 1. Currently, the patient is receiving treatment with insulin combined with oral hypoglycemic drugs, but blood glucose control is still poor, and the patient is intolerant to metformin hydrochloride. However, after fenofibrate treatment (200 mg/day), the blood lipid levels were significantly reduced compared with the initial diagnosis.

**Table 1:** Treatment and follow‐up results

	BG (mmol/L)	HbA1c (%)	CP (0–60–120 min) (ng/mL)	TG (mmol/L)	TC (mmol/L)	LDL‐c (mmol/L)	HDL‐c (mmol/L)	UA (μmol/L)
Day 0	Glargine 20 units/day + RI 40 units/day							
	20.7	15.9	1.14–5.18–5.51	49.28	25.63	16.74	0.34	766.2
Day 11	Glimepiride 2 mg/day + Acarbose 50 mg/tid + Fenofibrate 200 mg/day							
	–	–	–	7.47	3.06	1.41	0.55	–
Day 205	Acarbose 50 mg/tid + Pioglitazone hydrochloride 30 mg/day + PHI 10 IU/day + RI 30 unit/day + Fenofibrate 200 mg/day							
	15.9	10.6	2.53–3.32–3.98	4.52	3.16	1.31	0.66	494.6

BG, blood glucose; CP, serum C‐peptide; HbA1c, glycosylated hemoglobin A1c; HDL‐c, high density lipoprotein cholesterol; LDL‐c, low density lipoprotein cholesterol; PHI, protamine zinc recombinant human insulin; RI, recombinant human insulin; TC, total cholesterol; TG, triglyceride; UA, uric acid.

## DISCUSSION

Laminopathy is a rare monogenic disease caused by mutations in the LMNA, LMNB, ZMPSTE24, and other genes that encode laminins^2^, ^3^. These abnormalities affect nuclear DNA replication, chromatin anchoring, spatial positioning of nuclear pore complexes, binding of nuclear membrane proteins, and nuclear stability^4^, ^5^. Currently, 273 LMNA gene mutation sites associated with laminosis have been identified, resulting in over 10 clinical phenotypes^6^.

This report describes a juvenile case of laminopathy with diabetic ketoacidosis at the onset. Genetic analysis and testing confirmed a sporadic case of LMNA(p.T10I) mutation. Clinical manifestations include progeria facies, generalized lipodystrophy, insulin‐resistant diabetes, severe fatty liver, muscle atrophy, and skin papulosis. Due to the lack of lonafarnib for the treatment of progeria and metreleptin for the treatment of lipodystrophy in China^7^, ^8^, patients can only be treated with hypoglycemic and lipid‐lowering drugs. Previous studies have reported the efficacy of rosiglitazone in reducing blood glucose and blood lipid in patients with lipoatrophic diabetes mellitus^9^, ^10^. In this patient, fenofibrate therapy reduced triglyceride levels from 49.28 to 4.52 mmol/L after 6 months. The patient's HbA1c levels remained as high as 10.6% after 6 months of treatment with insulin (40 units/day), pioglitazone hydrochloride (30 mg/day), and acarbose (150 mg/day). The patient had shown significant abnormalities in the growth and development of adipose tissue, muscle, bone, and skin systems in childhood, and had not been diagnosed, suggesting an insufficient understanding of laminopathy among clinicians.

Diagnosing laminopathy requires three features^11^: firstly, the affected tissues are derived from the mesoderm (including skin, skeletal muscle, adipose tissue, bone, and myocardium); secondly, the affected tissues are either stunted or have accelerated degeneration; and thirdly, there is a molecular diagnostic basis related to lamin deficiency. The clinical manifestations of laminA/C patients are diverse, indicating that laminA/C protein expression may vary across cells and have multiple functions. In 2004, Csoka *et al*.^12^ reported the first case of Seip syndrome caused by a p.T10I mutation in LMNA gene. The patient presented with hyperlipidemia, hyperglycemia, subcutaneous fat atrophy, and premature aging. It is proposed that mutations at different sites of the LMNA gene can affect the combination of chromosomes and transcription regulatory factors by changing the tertiary structure of lamin A protein, leading to various clinical manifestations. In 2008 and 2012, Mory *et al*.^13^, ^14^ reported two cases of generalized lipodystrophy caused by the p.T10I mutation. In 2017, Hussain *et al*. analyzed the clinical data of 10 patients with the p.T10I mutation and shared common clinical characteristics. Therefore, it was recommended that patients with the p.T10I mutation be considered as a subtype of laminopathy called generalized lipodystrophy‐associated progeroid syndrome.^1^ The mutation and clinical manifestations of the patient in this case were consistent with those of previously reported GLPS patients, further confirming the patient's clinical and molecular diagnosis. However, limited medical resources led to some gaps in the diagnosis and treatment of this patient, such as the lack of detection of leptin levels and the use of dual‐energy x‐ray to determine total body fat content and distribution and the absence of etiological treatments.

## DISCLOSURE

The authors declare no conflict of interest.

Approval of the research protocol: N/A.

Informed consent: All procedures performed in this study were in accordance with the ethical standards of the institutional and/or national research committee(s) and with the Helsinki Declaration (as revised in 2013). Written informed consent was obtained from the patient for publication of this case report and accompanying images.

Registry and the registration no. of the study/trial: N/A.

Animal studies: N/A.
